# Risk factors for rapid progressive neurological deterioration in patients with cervical spondylotic myelopathy

**DOI:** 10.1186/s13018-021-02227-6

**Published:** 2021-01-21

**Authors:** Weiyang Zhong, Lin Wang, Tianji Huang, Xiaoji Luo

**Affiliations:** Department of Orthopedic Surgery, The First Affiliated Hospital of Chongqing Medical University, Chongqing, 400016 P. R. China

**Keywords:** Cervical spondylotic myelopathy,, Risk factor,, MR T2-hyperintensity

## Abstract

**Background:**

The rapid progressive cervical spondylotic myelopathy (rp-CSM) which had a course of CSM less than 1 month and suffered rapidly progressive neurological deterioration had few reports. Therefore, it is important for us to recognize the pathophysiology of CSM especially the rp-CSM. The study aimed to investigate the risk factors for rapidly progressive (rp) neurological deterioration in patients with cervical spondylotic myelopathy (CSM).

**Methods:**

A total of 159 patients were reviewed and divided into an rp-CSM group and a chronic-CSM (c-CSM) group. Various clinical indexes, including age, sex, Japanese Orthopaedic Association (JOA) score, intramedullary MR T2-hyperintensity, congenital/degenerative spinal stenosis, and local type of ossification of the posterior longitudinal ligament (OPLL), were analyzed, and independent risk factors were investigated.

**Results:**

Thirty-four of 159 patients (21.4%) were diagnosed with rp-CSM. All patients were followed up for a mean of 68.56 ± 14.00 months in the rp-CSM group and 62.66 ± 19.95 months in the c-CSM group. No significant difference was found in sex, mean age, smoking and drinking status, diabetes mellitus (DM), hypertension, surgery time, blood loss, JOA score, degenerative spinal stenosis, or OPLL (local). Univariate analyses demonstrated that rp-CSM patients tended to have MR T2-hyperintensity, longer hospital stay, shorter waiting time for surgery, more congenital spinal stenosis, and worse neurological function and to prefer more posterior surgeries than c-CSM patients. A multiple logistic regression analysis showed that congenital spinal stenosis and MR T2-hyperintensity were independently related to the presence of rp-CSM.

**Conclusions:**

MR T2-hyperintensity and congenital spinal stenosis were risk factors for rp-CSM. Although neurological function deteriorates rapidly, early surgical decompression is recommended and can achieve good neurological recovery after surgery, indicating that rp-CSM could be a reversible condition.

## Background

Cervical spondylotic myelopathy (CSM) is usually considered a slow, progressive spinal disease that is a major cause of spinal cord dysfunction, particularly in elderly individuals, but it is often delayed to diagnosis. Not all patients with presence of spinal cord compression have symptoms, and the progression ofCSM varies by patients. Magnetic resonance imaging (MRI) is an important imaging choice for patients with suspected CSM and the patients suspected of CSM is recommended to spine surgeon immediately and the severe patients can be typically treated surgically [1–3].

However, we focused on chronic year-by-year neurological exacerbation of CSM. A few patients with CSM suffered rapidly progressive neurological deterioration, particularly with walking disorders. Compared with chronic CSM (c-CSM), there have been few reports on the characteristics of rapidly progressive CSM (rp-CSM) [4, 5]. Therefore, it is important for us to recognize rp-CSM and the pathogenesis of rp-CSM. This study investigated the characteristics and potential risk factors for rp-CSM.

## Material and methods

### Population selection

This study was approved by the Institutional Review Board of our hospital and was conducted according to the principles of the Declaration of Helsinki. All the patients provided their written informed consent to participate in our study prior to the storage of their data in the hospital database. The cases of patients visiting our hospital between January 2010 and January 2014 were retrospectively reviewed. rp-CSP was defined as when the patients had a course of CSM less than 1 month and suffered rapidly progressive neurological deterioration, causing difficulty in maintaining a standing posture or walking without support [4, 5]. c-CSM was defined as when the patients had a course of CSM of more than 1 month [4, 5]. Inclusion criteria: severe patients who failed conservative treatment, suffered rp-CSM or c-CSM, and underwent surgery 3–4 days after hospitalization. Exclusion criteria include metastatic fractures, primary tumors, a history of any cervical spine surgery, high-energy trauma fractures, a history of cervical trauma, or infections.

### Surgical technique

All surgeries were performed by the same senior spinal surgeon. Anterior surgeries included anterior cervical discectomy and fusion (ACDF), anterior cervical corpectomy and fusion (ACCF), and hybrid procedures. Posterior surgeries included open-door laminoplasty or laminectomy and fusion. Postoperatively, all patients were required to wear a Philadelphia hard cervical collar for 6 to 8 weeks. In addition, the patients began step-by-step training of their neck muscles.

### Outcome assessment

The neurological status was evaluated preoperatively and at the final follow-up (FU) based on the Japanese Orthopaedic Association (JOA) score. The affected level of intramedullary hyperintensity lesions was assessed on a magnetic resonance imaging (MRI) T2-weighted system (Siemens, Germany). Congenital spine stenosis was assessed by the Torg-Pavlov ratio, which was defined as follows: on the X-ray, the ratio of the sagittal diameter of the spinal canal to the sagittal diameter of the vertebral body was less than 0.75, while the Palvov value of degenerative spine stenosis was more than 0.75 and less than 1.0.

### Statistics

The statistical analysis was performed using univariate analyses to compare the preoperative and postoperative outcomes of the two groups, and a multivariate logistic regression analysis was performed to determine the factors associated with the presence of rp-CSM using the Statistic Analysis System (SAS Institute Inc., Cary, NC, USA). The results are expressed as the group means±SD. Differences with a *P* value < 0.05 were considered significant.

## Results

Thirty-four of 159 patients (21.4%) were diagnosed with rp-CSM. All patients were followed up for a mean of 68.56 ± 14.00 months in the rp-CSM group and 62.66 ± 19.95 months in the c-CSM group (*P* > 0.05). No significant difference was found in sex, mean age, smoking and drinking status, diabetes mellitus (DM), hypertension, surgery time, blood loss, JOA score, congenital/degenerative spinal stenosis, or OPLL (local) (*P* > 0.05) between the groups. A comparison of the surgical approaches showed that there was no significant difference between anterior surgery and anterior combined with posterior surgery except the posterior approach (Table 1).

**Table 1:** Clinical information between groups

	rp-CSM	c-CSM	*P* value
No. of patients (*n*)	34	125	
Males/females (*n*)	19/15	68/57	0.8786
Mean age	57.24 ± 11.86	56.51 ± 11.64	0.7494
Smoker(%)	32.4%	16.8%	0.1286
Drink (%)	23.5%	17.6%	0.6589
Diabetes mellitus (%)	11.8%	10.4%	0.8208
Hypertension (%)	35.3%	15.2%	0.1020
Hospital stay	15.97 ± 6.92	12.78 ± 4.67	0.0019
Waiting period to surgery (m)	0.94 ± 0.43	25.80 ± 35.24	< 0.0001
Blood loss (ml)	160.0 ± 214.0	113.3 ± 108.8	0.0814
Surgery time (min)	158.4 ± 58.39	145.7 ± 57.66	0.2568
MR T2-hyperintensity (%)	88.2%	24.8%	< 0.0001
Congenital spinal stenosis (%)	8.8%	10.4%	0.7881
Degenerative spinal stenosis (%)	70.6%	56.8%	0.1479
OPLL (local) (%)	5.9%	7.2%	0.7900
JOA			
Pre-operation	14.00 ± 1.97	13.94 ± 1.82	0.8760
Final FU	16.62 ± 0.78	16.57 ± 0.75	0.7360
Surgery approach			
Anterior surgery	76.5%	90.4%	0.0689
Posterior surgery	20.6%	8.8%	0.0188
Anterior + posterior surgery	2.9%	0.8%	0.3236
Mean FU (m)	68.56 ± 14.00	62.66 ± 19.95	0.1076

Univariate analyses demonstrated that rp-CSM patients tended to have MR T2-hyperintensity (rp-CSM 88.2% vs. c-CSM 24.8%; *P* < 0.0001), longer hospital stay (rp-CSM 15.97 ± 6.92 days vs. c-CSM 12.78 ± 4.67 days; *P* = 0.0019), shorter waiting time for surgery (rp-CSM 0.94 ± 0.43 m vs. c-CSM 113.3 ± 108.8 m; *P* < 0.0001), more congenital spinal stenosis (rp-CSM 48.8% vs. c-CSM 10.4%; *P* = 0.0181), and worse neurological function (JOA: rp-CSM 8.73 ± 1.73 vs. c-CSM 13.94 ± 1.82; *P* < 0.0001) and to prefer more posterior surgeries (rp-CSM 20.6% vs. c-CSM 8%; *P* = 0.0188) (Table 1).

**Fig. 1:**
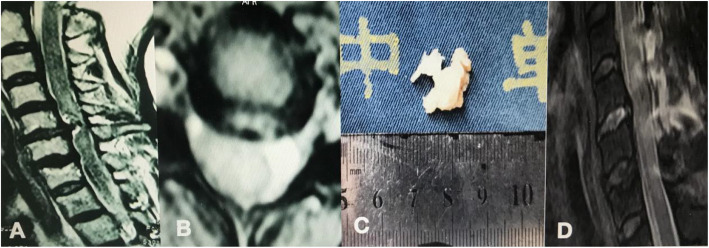
A 66-year-old female patient presented with Brown-Sequard syndrome of the left limbs and trunk for 3 days and was diagnosed with rp-CSM. **a**, **b** MRI showed spinal compression and T2-hyperintensity at the C5/6 disk level with edema at the C4–5 and C5–6 levels, probably due to spinal cord ischemia. **c** The patient underwent laminectomy and fusion, and the large disk was removed. **d** Postoperative MRI showed that decompression was satisfactory and neurological symptoms improved significantly

A multiple logistic regression analysis showed that congenital spinal stenosis (odds ratio (OR) = 3.95, 95% confidence interval (CI) 1.30–17.05, *P* = 0.030) and MR T2-hyperintensity (OR = 12.3, 95% CI 1.49–78.94, *P* = 0.021) were independently related to the presence of rp-CSM (Fig. 1).

## Discussion

With the increasing incidence of CSM, the clinical presentation and natural history of CSM vary, and CSM carries a high risk for disability [1–3, 6–10]. The aim of MRI is to help diagnose and manage patients earlier, especially those with rp-CSM [11]. In our study, 88.2% of rp-CSM patients presented with MR T2-hyperintense lesions, which was a risk factor, as reported previously by Morishita et al. [4, 5, 10–13]. On MRI, T2-hyperintense changes are considered to reflect the pathologies of spinal cord injury, such as edema, gliosis, demyelination, and myelomalacia. The high frequency of MR T2-hyperintensity might be indicative of spinal edema followed by acute spinal ischemia. This finding suggested the existence of microvascular lesions, causing glucose dysfunction and insufficient blood supply in the cervical spinal cord, which ultimately resulted in acute spinal cord ischemia observed as MR T2-hyperintensity. Congenital spinal stenosis, as a risk factor, could reduce the volume of the spinal canal, which could aggravate spinal cord injury. More attention should be paid to CSM patients with congenital spinal stenosis who could suffer rapid progressive neurological deterioration, and strict follow-up is recommended.

It was reported that there is a relationship between diabetes and a poor outcome in patients with CSM and that microvascular disorder caused by DM pathophysiology could affect or aggravate CSM [14, 15]. However, in our study, DM, smoking status, and hypertension were not related to rp-CSM. We agree with the coexistence of comorbidities that could be a potential risk of microvascular dysfunction and could not fully explain and understand the pathophysiological condition in patients with CSM, especially rp-CSM. More basic experiments are needed to investigate the pathogenesis of rp-CSM.

In the patients with rp-CSM and c-CSM, there was a significant difference in preoperative JOA score, and the neurological function of rp-CSM was much worse than that of c-CSM. The JOA score at the final FU was significantly improved, but there was no significant difference between the groups. The surgical treatments were both effective for rp-CSM and c-CSM patients. Furthermore, the rp-CSM patients showed good neurological recovery after surgery, indicating that this condition could be reversible. The optimal surgical timing and approach are still controversial and can vary depending on multiple factors, such as the location of the spinal cord compression, affected levels, and patient comorbidities [12, 13, 15–19]. In our study, rp-CSM patients had a shorter waiting period to surgery because of rapidly progressive neurological deterioration, and earlier decompression was considered. According to the direction of spinal cord compression and the number of surgical segments, different surgical approaches were preferred. In our study, in both groups, more patients were performed by the anterior approach without significant difference. The rp-CSM patients had spinal stenosis performed by posterior surgeries, which had longer hospital stays, while more anterior surgeries were performed inc-CSM patients. Regardless of the surgical approach, early decompression could achieve good neurological recovery without missing the window of opportunity for surgery.

However, our study had a few limitations. First, the retrospective nature of the small-sample study may be associated with bias. Second, the definition of rp-CSM was clinically discussed, but more clues regarding biomechanics, cytology, and pathology are needed. The timing of surgery for rp-CSM patients was difficult to determine, but it mostly depended on the neurological status and on the surgeon’s management or experience. Third, our study investigated whether MR T2-hyperintensity was associated with rp-CSM. T1-weighted, T2-weighted, or STIR sequences of the MR signal changes will be studied to gain more understanding of rp-CSM. In the future, prospective, randomized, grouped studies with long-term follow-up periods are needed.

## Conclusion

In our study, MR T2-hyperintensity and congenital spinal stenosis were risk factors for rp-CSM. Although the neurological impairment deteriorated rapidly, early surgical decompression is recommended and could achieve good neurological recovery after surgery, indicating a reversible condition.

## Data Availability

The datasets generated and/or analyzed during the current study are not publicly available due to the data is confidential patient data but are available from the corresponding author on reasonable request.

## Abbreviations

CSM: Cervical spondylotic myelopathy
rp-CSM: Rapid progressive cervical spondylotic myelopathy
C-CSM: Chronic-CSM
JOA: Japanese Orthopaedic Association
OPLL: Ossification of the posterior longitudinal ligament
ACDF: Anterior cervical discectomy and fusion
ACCF: Anterior cervical corpectomy and fusion
DM: Diabetes mellitus
